# Radical Decisions in Cancer: Redox Control of Cell Growth and Death

**DOI:** 10.3390/cancers4020442

**Published:** 2012-04-25

**Authors:** Rosa M. Sainz, Felipe Lombo, Juan C. Mayo

**Affiliations:** 1 Cellular Biology and Morphology Department, School of Medicine, University of Oviedo, Oviedo 33006, Spain; E-Mail: mayojuan@uniovi.es; 2 University Institute of Oncology of Asturias (IUOPA), University of Oviedo, Oviedo 33006, Spain; E-Mail: lombofelipe@uniovi.es; 3 Functional Biology Department, Area of Microbiology, School of Medicine, University of Oviedo, Oviedo 33006, Spain

**Keywords:** redox control, proliferation, survival

## Abstract

Free radicals play a key role in many physiological decisions in cells. Since free radicals are toxic to cellular components, it is known that they cause DNA damage, contribute to DNA instability and mutation and thus favor carcinogenesis. However, nowadays it is assumed that free radicals play a further complex role in cancer. Low levels of free radicals and steady state levels of antioxidant enzymes are responsible for the fine tuning of redox status inside cells. A change in redox state is a way to modify the physiological status of the cell, in fact, a more reduced status is found in resting cells while a more oxidative status is associated with proliferative cells. The mechanisms by which redox status can change the proliferative activity of cancer cells are related to transcriptional and posttranscriptional modifications of proteins that play a critical role in cell cycle control. Since cancer cells show higher levels of free radicals compared with their normal counterparts, it is believed that the anti-oxidative stress mechanism is also increased in cancer cells. In fact, the levels of some of the most important antioxidant enzymes are elevated in advanced status of some types of tumors. Anti-cancer treatment is compromised by survival mechanisms in cancer cells and collateral damage in normal non-pathological tissues. Though some resistance mechanisms have been described, they do not yet explain why treatment of cancer fails in several tumors. Given that some antitumoral treatments are based on the generation of free radicals, we will discuss in this review the possible role of antioxidant enzymes in the survival mechanism in cancer cells and then, its participation in the failure of cancer treatments.

## Abbreviations

γ-GCS: γ-Glutamyl Cysteine Synthase
OH: Hydroxyl radical
8OHdG: 8-hydroxy-2'-deoxyguanosine
ASK: Apoptosis Signal-regulated kinase 1
cGMP: cyclic Guanosyl Monophospate
CuZnSOD (SOD1), Cu: Zn-dependent Cytoplasmic Superoxide Dismutase
CYP: Cytochrome P450
EGF: Epithelial Growth Factor
EPA: Eicosapentaenoic acid
GC: Guanylate Cyclase
GSH: Glutathione
GSTs: Glutathione S-Transferases
HO-1: Heme Oxygenase 1
HPLC: High Pressure (performance) Liquid Chromatography
HSF1: Heat Shock Factor 1
MAPKs: Mitogen-activated Protein Kinases
MnSOD (SOD2): Manganese-dependent Mitochondrial Superoxide Dismutase
MRP1: Multidrug Resistant Protein-1
mTOR: *mammalian target of* R*apamycin*
NER: Nucleotide Excision Repair
NES: Nuclear Export Signal
NFκB: Nuclear factor-κB
NOS: Nitric Oxide Synthase
O_2_**^•−^**: Superoxide radical
OGG1: 8-oxo-guanine DNA Glycosylase
OxyR: Hydrogen peroxide-inducible genes activator
PARP: polyadenosine-5'-dephosphate ribose polymerase
PDGF: Platelet-derived Growth Factor
PDGFR: Platelet-derived Growth Factor Receptor
PI3K: Phosphatidyl Inositol 3-Kinase
RNS: Reactive Nitrogen Species
ROS: Reactive Oxygen Species
RPTKs: Receptor-coupled Protein Tyrosine Kinases
SAPK**/**JNK: Stress-activated Protein Kinases
SNPs: Single Nucleotide Polymorphism
TNF: Tumor Necrosis Factor
TRAF1: TNF Receptor-associated Factor 1
Trx: Thioredoxin
VEGF: Vascular Endothelial Growth Factor
VSMCs: Vascular Smooth Muscle cells
XOR: Hypoxanthine-Xanthine Oxidoreductase
Yap1: Yeast-activating Protein 1

## 1. Introduction

Cancer cells are continuously dividing and avoiding any control on their cell cycle. Therefore seeking those mechanisms altered and also, molecules able to control its progression has attracted the attention of scientists. It is reasonable to admit that there is not only one way to explain why cancer cells behave as they do. Moreover, cancer cells show a complete alteration of their physiology, metabolism, genome and proteome but, that only partially explains their behavior. Once we admit that not a few mutations, proteins, pathways or phenomenons are changed and also, that those mutations, proteins, pathways or facts can change in a precise cell type but cannot be changed in another cell types, then we will be able to understand at least in part what a cancer cell represents. Several changes, endogenous and exogenous, alter the physiology of normal cells and convert them in a way difficult to understand and manage.

Then, cancer must be considered a complex and multifaceted illness that affects different patients in various ways. Cancer evidences the imbalance of control mechanisms that assure the proper functioning of cells. Looking inside the complex machinery that assures our body functioning, it is not difficult to imagine that simple changes can promote imbalances and a great disaster. Not only mutations, but also DNA repair mechanisms, must protect our DNA from damage. Not only growth factor signaling, but also feedback mechanisms, protect us from continuous stimulation of cell growth. In the same way, not only oxidative phosphorylation, but also antioxidant mechanisms protect our macromolecules from the damage caused by toxic byproducts.

Nowadays, the search for the alteration of complete pathways and personalized therapies seem to be the most appropriate way to fight against this disease. Cancer is a leading cause of death worldwide and kills more than a 500,000 persons year only in America, below only heart diseases. It accounted for 7.9 million deaths worldwide (around 13% of all deaths) in 2007. Although anyone can develop cancer, about 78% of all cases are diagnosed in people 55 years old and older, but the most frequent types differ between men and women. Lung, stomach, liver, colon and breast cancer cause the most cancer deaths each year. Deaths from cancer worldwide are projected to continue rising, with an estimated 12 million deaths per year expected by 2030.

Tumor formation is a multistage process involving a series of events which include the accumulation of genetic and epigenetic alterations leading to the progressive transformation of a normal cell into a malignant one. Modifications may be started by external agents and inherited genetic factors. Cancer cells acquire abilities that normal cells do not have, including survival mechanisms which enable them to evade cell death [1]. Despite the development of new therapies, cancer is still the second leading cause of death in Western countries. Several types of tumors develop a resistance to avoid cell death induced by conventional treatments.

A free radical is defined as an atom or a molecule with one or more unpaired electrons in an outermost valence shell. ROS include O_2_-derived free radicals, in fact O_2_ is a biradical but also, non-radical derivatives of O_2_ such as hydrogen peroxide H_2_O_2_ exist. ROS have been normally recognized by radiation chemists as products generated by the radiolysis of water, and today this is the principal mechanism employed by radiologists to treat cancer [2]. Endogenous radical production can occur and approximately 2% of the total mitochondrial O_2_ consumption results in O_2_^−^ production [3], in addition to the ROS formed as byproducts of certain enzymatic reactions [4,5]. For this reason cells must maintain an endogenous antioxidant capacity based on water or lipid soluble compounds and enzymatic systems that remove ROS but also, may include proteases specialized in the removal of oxidatively modified proteins, DNA or lipids [6].

An increase in free radicals, through an increase in production or a defect in removal, causes DNA, protein or lipid damage that can play a critical role in carcinogenesis but also, a delicate change in redox conditions can play a role in tumor progression by affecting cell growth or survival. In this review, we will go over the main points that show the critical role of free radicals, antioxidants and redox balance in cell growth and survival. The review will focus attention on recent reports and the important role, not only as carcinogens, but also as cell signaling molecules of oxygen and nitrogen radicals. Also, the main role of antioxidant enzymes in cancer progression as well as its particular importance in cell survival in non-pathological and pathological conditions will be discussed below.

## 2. Free Radicals, Oxidants and Antioxidants

### 2.1. Importance and Source of Free Radicals in Living Cells

Several decades ago an inverse correlation between the rate of oxygen consumption and the longevity of mammals was proposed. Although no longer accepted, this “rate-of-living” theory of aging highlighted the importance of oxygen metabolism in eukaryotic cells [7]. Later on, more than five decades ago, Harman proposed the free radical theory which states that oxygen and nitrogen free radical species (ROS/RNS) are formed endogenously, being hydroxyl (^•^OH) and peroxyl radicals (^•^O_2_H) the major “culprits” of biological oxidative damage. Furthermore the free radical theory relates them directly with the aging process [8]. Earlier discoveries pointed out the importance of oxygen toxicity by demonstrating the inhibition of several enzymes [9]. However, initially this theory did not attract much of attention from scientists, until a parallel was demonstrated between the effects of O_2_ and those of ionizing radiation, leading to the discovery that oxygen free radicals actually occur in biological systems and are responsible for most of the damaging effects observed [10,11]. Interestingly, now it is perfectly established that oxygen radicals are also derived from normal cell metabolism as a side product.

Apparently *in vivo* ROS/RNS can be derived from many different sources, including auto-oxidation, photochemical or enzymatic reactions and may involve both endogenous compounds and various xenobiotics. Among enzymes shown to be capable of generating ROS, cytochromes P450, oxidases, peroxidases, lipoxygenases and dehydrogenases are included [12]. The involvement of xenobiotics can be particularly important in determining the extent of ROS generated by these enzymes, relating them directly with mutagenesis (see below). However, among all the potential sites of ROS production *in vivo*, the main source is the electron transport chains, especially those present in bacterial membranes or in the endoplasmic reticulum and mitochondria in eukaryotic cells. Thereby this latter organelle is the main ROS source [13,14,15,16], which actually prompted Harman in 1972 to propose a slight “fine tuning” of the free radical theory, focusing the attention on mitochondria [17]. Based on several reports it has been suggested that even at physiological O_2_ concentrations, occasional electron leakage occurs along mitochondrial respiratory chain-especially at earlier components such as respiratory complex I, leading to a roughly 1–3% of O_2_^•−^ formation. Eventually this amount would increase at elevated O_2_ concentrations [18]. Although the quantity produced *in vivo* under both normal and pathologic conditions is unknown, some authors claim that the actual production of radicals by intact mitochondria in non-pathologic tissues is likely to be less than 2% under normoxic conditions. However, substantially more may be produced under damaging conditions or in the presence of numerous xenobiotics and carcinogens.

Based on this evidence, under basal conditions superoxide radical (O_2_^•−^) and H_2_O_2_—which is not a free radical *per se*—account for the majority of ROS generated inside cells. Most authors agree that, unless some cellular defensive mechanism(s) comes to the rescue, mitochondrial activity would eventually lead to a rise in intracellular O_2_^•−^ concentration over a threshold, thus compromising cell survival [18]. Remarkably O_2_^•−^ when compared to other free radicals, seems to be innocuous from the reactivity point of view. Nonetheless its protonated form, HO_2_^•−^, is more reactive and can cross cell membranes as H_2_O_2_ which is considerably more dangerous. However, again H_2_O_2_ is not highly reactive from the chemical point of view but it is well demonstrated that indirectly it can lead to the formation of other highly-damaging free radicals through the well-known Haber-Weiss or more frequently the iron-catalyzed Fenton reactions. The question is how cells counteract this continuous “radical” challenge. For this purpose, several antioxidant mechanisms, either endogenous enzymatic and non-enzymatic as well as exogenous small molecular weight antioxidants from diet, appeared during evolution as essential pieces to prevent ROS/RNS-mediated cell death.

### 2.2. ROS/RNS and Oxidative Stress

Conceptually speaking, once it was assumed that endogenously formed radicals from normal metabolic processes could play a role in aging an in many other diseases and that, fortunately, there is a set of intracellular antioxidant mechanisms confronting them inside cells, Sies first introduced the term “oxidative stress” almost 30 years ago [19]. Later in 1991 he exactly defined the term as “a disturbance in the prooxidant-antioxidant balance in favour of the former, leading to potential damage” which is usually called oxidative damage. Consequently, at least in a healthy situation, there must be a necessary balance between production of ROS/RNS and their clearance by antioxidants. Several situations could then contribute to oxidative stress, namely (i) diminished antioxidants, e.g., reduction in antioxidant defenses like antioxidant enzymes; (ii) increased production of ROS/RNS, e.g., elevation of O_2_ or presence of radical-forming xenobiotics, toxins, *etc*. Accordingly the term “mitochondrial oxidative stress theory of aging” it is now commonly accepted to redefine the free radical theory to explain aging. Moreover, several lines of evidence strongly support the instrumental role of oxidative stress, not only in aging but also in many other diseases, including cancer [20,21,22]. Nevertheless, along the evolutionary process, eukaryotic cells developed a reduction-oxidation (redox) regulatory system involving basal levels of free radicals in such way that those molecules became important signaling effectors as a basic redox control tool, joining the two other common mechanism of cell signaling, *i.e*., high-energy chemicals and transmembrane ion-gating mechanisms [23,24].

## 3. Cell Damage by Free Radicals in Normal Tissues

### 3.1. Basal Levels of Free Radicals

Thousands of studies have already shown that free radicals *per se*, due to their reactive chemical nature can harm multiple intracellular targets, including major macromolecules (*i.e*., proteins, DNA or lipids). Additionally, free radical species indirectly formed by the action of xenobiotics, pollutants or other carcinogens are indeed responsible for oxidative damage of macromolecules that finally ends up in nucleotide mutations in DNA. Moreover, during the past two decades extensive research has revealed the role of oxidative stress in causing chronic inflammation which is directly linked to chronic diseases like cancer [25]. In this way Halliwell has elegantly addressed how redox control in an increasing oxidative situation might induce one of the following outcomes when cells move progressively from a highly reduced to a highly oxidized intracellular environment: (1) increased proliferation; (2) adaptation by up-regulation of antioxidant defense; (3) cell injury; (4) senescence; (5) cell death [26].

Usually resting cells show a highly reduced intracellular environment, rich in reduced glutathione (GSH) with high concentrations in other endogenous antioxidants like ascorbate and with a ratio GSH: GSSG over 99% [18]. However at this stage, some degree of oxidized microenvironments (*i.e*., endoplasmic reticulum) is needed, which allow folding of newly formed proteins to form disulfide bonds [27]. As it will be discussed below, several transcription factors are controlled by redox signaling. Consequently, mitochondrial or NAD(P)H oxidase ROS production, usually through final generation of low, non-toxic, basal levels of H_2_O_2_ regulates several transcription factors that stimulate the proliferation of several cell types in culture [28,29]. Furthermore, exogenously added hydrogen peroxide is able to induce cell proliferation in certain cell types [30,31,32]. Likewise contact growth inhibition observed in cultured cells is due to a decrease of intracellular ROS production [33]. If either external source of ROS persists or there is an overproduction of radicals inside cells, oxidative damage eventually rise and cells enter into next stage of adaptation, which includes, among others, up-regulation of antioxidant enzymes. This process may also occur in early stages of tumor development and it also appears during ROS generating treatments like radiation or chemotherapy, indicating a critical role of antioxidant enzymes, specially MnSOD, in such radiation-induced adaptation [34,35,36]. Finally in a last stage, along with an increase in oxidative stress, cells would enter into a cell death-inducing situation.

While redox control of cell proliferation has only received attention in the last few years, the ROS/RNS-mediated damage is much better known. When chemical or physical stimulus continues, cell injury and/or senescence occur. In a certain range of oxidative stress there is still a reversible response and cell might return situation to normal but once a critical oxidative damage is reached, cell death becomes inevitable. Thus apoptosis is usually triggered by oxidizing agents and subsequently antioxidants will shut down free radical-mediated oxidation. Among other apoptosis-related factors, caspases are notably dependent on certain sulfhydryl residues. Consequently, if intracellular environment is too oxidized, caspases do not work properly and apoptosis is blocked [37]. This helps to explain the pro-apoptotic effects of some antioxidants, especially in combination with other agents [38].

### 3.2. DNA Damage Caused by Free Radicals and Its Role in Carcinogenesis

ROS/RNS can be harmful to most macromolecules, including lipids, proteins or DNA [18]. When oxidative stress occurs, it may be difficult to establish which of these molecules reacts first with ROS/RNS. However oxidative stress itself would accelerate the normal spontaneous chemical decomposition suffered by DNA bases. Similarly, it is widely reported that DNA damage occurs continuously, measured as 8-hydroxy-2'-deoxyguanosine (8OHdG) or as other base modifications [39,40]. These modifications usually can be easily monitored by quantitative HPLC assays [41] and they can anticipate the presence of mutations as Cheng *et al.* demonstrated. Thus, 8OHdG causes G→T and A→C substitutions [42] and its presence has been associated with several types of tumors [43,44,45,46] but not with others [47]. Also, ROS/RNS generated by UV radiation induce tandem mutations in p53 in skin cancers [48]. From all ROS generated inside cells, only the highly reactive ^•^OH has energy to modify DNA [49] while other can participate through metal-catalyzed Fenton reactions giving rise to ^•^OH. Other useful biomarkers of ROS/RNS-mediated damage in cancer samples are lipid peroxidation, specially assayed as the presence of isoprostanes/isofurans or as aldehydes [50,51,52] as well as protein oxidative damage, measured as nitration or nitrosation of certain residues like tyrosine or as carbonyl content [52,53].

Among exogenous sources of reactive oxygen species, ionizing radiation, environmental agents and therapeutic agents can be included. All of them are able to act as human carcionogens. Ionizing radiation has been found to induce cancer in several species and affects at all stages of carcinogenesis. Inflammatory cells including neutrophils, eosinophils and macrophages contribute to the cellular burden of ROS. Phagocytes produce ROS through NADPH oxidase, the enzyme that catalyzes the single electron reduction of oxygen to O_2_^•−^. ROS generated by this mechanism play an important role in cellular defense by killing bacteria but have also been involved in the development of tumors [54]. In fact, it is considered that infection and chronic inflammation can contribute to 1 out of 4 of all cancers diagnosed [55]. A sustained inflammatory microenvironment provides a constant supply of ROS/RNS that could contribute with cytokines, chemokines and growth factors, in altering cellular homeostasis and lead to genomic instability and to raise the risk of cancer development. *In vitro* and *in vivo* evidences implicate inflammation in altering multiple pathways related to cancer progression [56]. Thus, mutation studies have suggested that chronic oxidative stress is associated with carcinogenesis. For example, ulcerative colitis is linked with higher incidence of colorectal cancer or chronic gastritis due to infection with *Helicobacter pilori* and therefore might be responsible of higher incidence of gastric cancer [57].

Oxidative DNA damage is a major source of mutation and its frequency is estimated at 10^4^ lesions/cell/day [58]. The most extensively studied oxidative DNA damage is the rate of 8-hydroxydeoxy guanosine (8-OHdG), which is mutagenic in bacteria and mammalian cells. 8-OHdG is elevated in tumor cells and in animal models of cancer. Its stable conformation can pair with both cytosine and adenine. G:C or A:T transversion caused by 8-OHdG in the DNA template is commonly found in mutated oncogenes or tumor suppression genes [42]. During replication, ROS can also react with dGTP to form 8-OHdG which is also able to be incorporated into DNA opposite a template strand. In fact, 8-OHdG has been widely used as a biomarker of DNA oxidative damage and it is useful to evaluate the level of oxidative stress in a particular system. Also, RNS produce during chronic inflammation cause 8-nitroguanine which has been considered a mutagenic DNA lesion and leads to G→T transversions [54].

In addition to a promotion of mutagenesis and initiation of tumor progression some polymorphisms have been reported in oxidative-stress related genes, including carcinogen metabolizing genes which also contribute to their role in carcinogenesis. About 10 million polymorphisms (SNPs) exist in the human genome, and while some of these variations are silenced, a small proportion are found in coding or regulatory sequences and imply phenotypic changes. Among others oxidative stress related genes or DNA repair genes, including carcinogen metabolizing genes, present genetic polymorphisms that may explain individual differences in their expression or activity and its role in carcinogenesis. Cytochrome P450 (CYP) gene family, a phase I detoxifying enzyme, or glutathione-S-transferase, an element of phase II metabolic enzymes and an antioxidant enzyme, which are in charge of detoxifying environmental carcinogens, are reported to be polymorphic and those polymorphisms are related to human cancers [55,56]. Also, polymorphisms in DNA repair genes which are implicated in 8-OHdG, including base excision repair (BER) enzymes or 8-oxo-guanine DNA glucosylase (OGG1) in addition to nucleotide excision repair (NER) can contribute to its role in carcinogenesis. In fact, epidemiological studies investigating the association between SNPs of OGG1 have related the variant allele to a significantly increased risk of human cancers including lung, esophageal, prostate or gastric cancer [20,57].

ROS/RNS may have a triggering effect in cell damage. It has been suggested that tumors are in a “pro-oxidant” state generating more free radicals, which is usually not accompanied by up-regulation of DNA repair mechanisms. According to current knowledge, ROS/RNS can participate at multiple levels in carcinogenesis, either by direct effects on DNA or by modulating signaling pathways leading to increase in cell proliferation. Nonetheless direct role of ROS/RNS in carcinogenesis is mostly related to initiation and promotion, whereas rest of tumor stages can be indirectly related to free radicals through the chronic inflammation-related deregulation of cytokines or other factors released by macrophages [58].

## 4. Redox Control of Cell Growth

### 4.1. ROS/RNS as Cell Signaling Molecules

It has been historically assumed that ROS are tumor initiators simply by inducing genomic instability. However in 1995 Sundaresan *et al.* [59] showed that the stimulation of rat vascular smooth muscle cells (VSMCs) by platelet-derived growth factor (PDGF) transiently increased the intracellular concentration of H_2_O_2_, suggesting that H_2_O_2_ may act as a signaling transducer molecule, the role of oxygen-derived radicals in cellular signaling has been widely accepted. Since then, it is considered that ROS increase tumorigenesis not only by its carcinogenic or DNA damaging properties, but also by activating signaling pathways that regulate cellular proliferation, angiogenesis and metastasis [60]. These opening studies demonstrated that inhibiting the rise in ROS levels following ligand addition halts a variety of downstream pathways. Thus, depleting H_2_O_2_ by the addition of catalase to culture media, the ability of PDGF and also epithelial growth factor (EGF) to stimulate tyrosine phosphorylation does not occur [61]. However not only tyrosine kinases are regulated by ROS, also, several other molecules, related to cell cycle control or proliferation, are control by them.

Different levels of ROS can activate different signaling pathways; therefore low or transient levels of ROS can activate proliferative signals, while high levels normally reduce cell proliferation by activating damage or cell death-related signals. It has been demonstrated that ROS activate kinases and inactivate phosphatases but also, they activate protein serine/threonine kinases, small G proteins or transcription factors signaling. All that pathways are in different ways altered in cancer cells which makes cancer cells to lack there control of proliferation and differentiation.

### 4.2. Redox Control of EGF and PDGF Signaling

The role of O_2_^•−^ and H_2_O_2_ as mitogenic mediators is related to its ability to modulate receptor tyrosine kinases. Cell surface receptors for fibroblast growth factor (FGF), epidermal growth factor (EGF), vascular endothelial growth factor (VEGF), macrophage colony stimulating factor, hepatocyte growth factor, nerve growth factor or insulin-like growth factor cross the membrane with a single transmembrane pass, they have an extracellular ligand-binding domain and all them, have a cytoplasmic domain that upon activation transfer an ATP group to a selected tyrosine residues [62]. Tyrosine residues sensitive to phosphorylation might be part of other receptors or downstream proteins involved in cellular signaling.

EGF has four different receptors, ErbB1, ErbB3, ErbB4, which share more than 50% homology that when it binds to its ligand, is autophosphorylated and form dimers or oligomers. The activated receptor triggers intracellular signaling and promotes proliferation or cell migration [63,64]. Unexpectedly, the activation of human epidermoid carcinoma cells by EGF increases ROS production, in particular intracellular H_2_O_2_ level are increased after stimulation [65]. Molecular approaches showed that H_2_O_2_ generation requires the receptor kinase activity of the receptor but also, that an increase in H_2_O_2_ is necessary but not sufficient for the increase in the steady-state level of protein tyrosine phosphorylation and that the inhibition of protein tyrosine phosphatase activity may be required. Later, it was demonstrated that H_2_O_2_ causes the oxidation of a sulfhydryl group at the active site of protein tyrosine phosphatases and that inhibition is reversed by the presence of antioxidants [66]. Redox-sensitive transactivation of EGFR occurs through a proximal tyrosine kinase, c-Src, which is shown to be activated by oxidative stress in cultured cardiac myocytes [67]. Similarly, the Src family of protein kinases, including phosphoinositol 3-kinase (PI3K), the GTPase-activating protein Ras, Src homology 2 phosphatase 2 (SHP2) and phospholipase C-γ (PLC-γ), bind and phosphorylate seven auto-phosphorylation specific sites of the PDGF receptor [68,69]. The activation of PDGFR by binding PDGF homo or heterodimeric proteins increases O_2_^•−^ production in human aortic smooth muscle cells [70]. Also, PDGF-BB was shown to generate O_2_^•−^ in human lung fibroblast [71] and in bovine tracheal myocytes [72]. In both, the reported increase in O_2_^•−^, shortly after ligand binding, was not accompanied by NADH oxidase activation or H_2_O_2_ production. Moreover, cells overexpressing a mutant form of Ras protein failed to increase intracellular superoxide production in response to mitogenic stimuli. In that way, it was proposed that PDGF stimulated intracellular O_2_^•−^ production via Ras-dependent activation of a O_2_^•−^ generating oxidase/electron transferase and that, this mechanism may be a common signaling pathway in a number of RPTKs. PDGF receptor triggers intracellular signaling by recruiting members of the Src family of protein kinases. By expressing several mutants of PDFG receptor in HepG2 cells, it was shown that PDGF induced H_2_O_2_ production was blocked by PI3K inhibitors and by dominant negative mutants of Rac1 and Rho family GTPases [73]. More recently, the role of the cell adhesion receptor, syndecan-4, in regulating growth factor-induced ROS generation was studied. Rat embryo fibroblasts (REFs) overexpressing syndecan-4 exhibited increased ROS levels compared to control cells and a parallel enhancement of PDGF-induced MAP kinase activity suggesting that syndecan-4 regulates PDGF-induced MAP kinase activation by altering ROS generation too [74].

### 4.3. Redox Control of FGF and VEGF Signaling

Fibroblast growth factors (FGF) are 20 different proteins that bind to four different receptors (FGFR-1 to FGFR-4) in order to achieve diverse effects in a variety of cell types. FGF receptor has also a three extracellular Ig-like domains, a transmembrane domain and an intracellular tyrosine kinase domain [75]. FGF binding promotes receptor dimerization and induces receptor kinase activation, recruiting FGF receptor stimulates several proteins, Grb2, SOS and SHP2. This recruitment promotes the activation of Ras and leads to signaling through ERK MAP kinase pathway. FGF factors have several biological functions, including a potent role in growth and angiogenic activities which make them important players in wound healing [76] but they have also been reported to play a role in embryonic development [77]. H_2_O_2_ production after FGF-2 stimulation was demonstrated in chondrocytes [78] and it correlates with increased expression of c-fos. Antioxidants as *N*-acetylcysteine, inhibited FGF-2 induced c-fos mRNA, which confirms the role of ROS in mediating FGF-2 biological signals, at least in chondrocytes. Later on, FGF-2 was also demonstrated to increase shortly after binding to its receptor intracellular levels of O_2_^•−^ via Ras-dependent activation of a O_2_^•−^ generating oxidase/electron transferase [71].

Additionally ROS signaling increases the expression and levels of growth factors. That is the case of VEGF, which is a dimeric glycoprotein that interacts with three receptor kinases denoted VEGFR-1, -2 and -3, generally expressed in endothelial cells. VEGF receptor stimulation activates several downstream signaling pathways which promote differentiation, migration, survival and permeability of endothelial cells. VEGF is the target of several antitumor agents since it is related to angiogenesis in tumors [79]. H_2_O_2_ was shown to increase VEGF gene transcription through activation of oxidative stress inducible transcription factors such as NFκB or AP-1 and this induction can be blocked by the employment of antioxidants [80].

### 4.4. ASK as a Redox Sensor

Since ROS generation mediates the signaling pathways trigger by growth factors and tyrosine kinases or phosphatases, critical players in cell growth control, are regulated by redox reactions, it seems clear that ROS can be considered as secondary messengers in cell cycle regulation or at least, they play a key role, as other classical secondary messengers, in cell growth and death control. ROS can modulate protein function is by changing the oxidative state of cysteine residues present in the catalytic domain of proteins. Those modifications cause posttranscriptional changes that influence protein activity, turnover and subcellular trafficking but also, protein-protein interactions. Consequently, reactive modifications of cysteine in proteins implicated in cell fate is a transitory, specific and reversible signaling mechanism which resembles to other signaling molecules [81]. It is considered that almost 0.5% of all cysteine residues in the proteome are subjected to continuous and reversible oxidation-reduction and this redox control may regulate several aspects of the structure or function of eukaryotic cells [82]. In contrast to regulation by phosphorylation, ROS are small, diffusible and highly reactive molecules that can act at a distance and do not required the direct interaction of kinases and their substrate. However, oxidants also tend to be produced and metabolized locally [83].

Thus, some proteins, including apoptosis signal-regulated kinase 1 (ASK1), thioredoxin (Trx), peroxiredoxin family or calcineurin seem to be regulated by a redox mechanism [84,85,86,87]. In all that cases, oxidants may have direct effects on targets altering protein-protein interactions and regulating its enzymatic function. In the particular example of ASK1, a member of the family of mitogen-activated protein kinase kinase kinases that is involved in the activation of stress activated protein kinases (SAPK/JNK kinases) and p38, it forms a complex with Trx which inhibits its activity. ASK1 is activated in cells treated with inflammatory cytokines or when cells are exposed to stressors such oxidative stress or ER stress. ROS increment after tumor necrosis factor (TNFα) stimulation promotes ASK1 activation and induces apoptosis [88]. By using a dominant negative mutant form of ASK1 cell death induced by TNFα or oxidative stress is inhibited. Also, ROS such as H_2_O_2_ are the most potent activator the ASK1. In fact, anticancer drugs or irradiation trigger apoptosis in cancer cells through ROS-mediated ASK1 activation [89]. Trx was identified as a negative regulator of ASK1 which stays inactive in resting cells until treatment with TNFα, H_2_O_2_ or ROS donors when it is fully activated. Trx is a redox sensitive protein that possesses two cysteine residues within its active center. After oxidizing Trx, ASK1 switches from an inactive to an active form and activates the kinase signaling pathway. Thus, ASK1-Trx complex works as a redox sensor which communicates cellular redox state into kinase signaling pathways [88]. Also, other antioxidant proteins as peroxiredoxin family or superoxide dismutase 1 can regulate intracellular signaling molecules in a redox dependent manner [86,87].

### 4.5. Role of Cysteine Containing Proteins in Redox Sensing

Although, it is considered that under physiological conditions cysteines might be in their protonated form (SH), some proteins in mammalian cells present oxidized cysteines. Thus, reactive cysteines are inactivated to a sulfenic ion when reacted with oxidative reactants. That change is reversible by the action of antioxidant proteins including Trx or glutaredoxins, however, when oxidative stress lasts reactive cysteines are converted to an irreversible oxidized status, a sulfinic ion. This scenario represents a reversible, specific and transient mechanism of cell signaling where persistent inactivation of proteins must exist while continued high levels of oxidative stress occurs. Proteins such as tyrosine phosphatases have a reactive cysteine in their active site. Since oxidative stress inactivates tyrosine phosphatases it is likely that persistent oxidation of these cysteine residues play a critical role in kinase pathways, which are one of the principal players in cellular growth control [24,81]. Also, covalent addition of glutathione to reactive cysteins may be a redox-dependent signaling mechanism. This mechanism has been reported after EGF stimulation, when transient glutathiolation of PTP-1B phosphatase has been found [90].

Thus, mild oxidative stress due to physiological unbalances is responsible for regulatory processes and represents the way by which a redox signal can be transduced within the cell. It was firstly evidenced in bacterial transcription factors. Thus OxyR is a good example of a redox sensitive prokaryotic transcription factor. Specific cysteine residues respond to H_2_O_2_, and the activation of OxyR involves the formation of a disulfide bond between Cys 199 and Cys 208 [91]. As a result, a change in DNA binding specificity occurs and the recruitment of RNA polymerase is the final result of OxyR redox changes. Later on it was found that a single thiol residue is critical for OxyR activity and this can be differently oxidized depending on the extent of redox unbalance and the nature of oxidizing molecules [92]. OxyR is a model of direct and highly inducible regulation that participates in survival of bacteria to adverse environments by modifying directly gene transcription after binding. In yeast, oxidative stress does not alter directly gene expression but instead it alters compartment location of transcription factors after oxidative stress. That is the case of yeast activating protein 1 (Yap 1), a functional homolog of the bacterial OxyR. Yap1 contains a noncanonical leucine-rich nuclear export signal (NES) embedded with the *C*-terminal cysteine-rich domain. Oxidative stress results in a conformational change that impedes Yap1 export and leads the accumulation of the protein in nucleus, which results in its enhanced nuclear concentration and binding affinity to DNA [93].

Changes in intracellular redox state are also an additional signaling event in multicellular eukaryotes’ transcriptional activity. Some redox sensitive transcription factors have also been found in multicellular eukaryotes as p53, c-Jun/AP-1, nuclear factor-κB (NFκB) or heat shock factor 1 (HSF1). Some of these proteins are important regulators of cell cycle and apoptosis which control the transcription of critical proteins for cellular biology. Some reports indicate that cells treated with oxidants or oxidative-stress inducing agents are efficiently activated for nuclear translocation. However, the binding of transcription factors to DNA is allowed only upon reduction. Reduction of Cys 62 of p50 subunit of NFκB must be maintained to assure a correct binding to the promoter regions of its target genes [94]. The nucleus is in fact a very reducing compartment, in which GSH and Trx are present at high concentrations as well as redox sensitive factors such as Redox factor-1 (Ref-1). Trx in turn translocates into nucleus and modifies cysteine residues of sensitive transcription factors. Reducing nuclear environment not only activated gene transcription after redox modifications of transcription factors but also, protects DNA from oxidative damage [95].

Considering all of the above, the modification of the redox state of cysteine residues of specific, redox-sensitive proteins, including transcription factors, mitogen activated protein kinases or phosphatases, might be a widespread mechanism in the regulation of protein function, since growth factors stimulate ROS production to activate protein tyrosine kinases or inactivate protein tyrosine phosphatases through a reversible, specific and transient mechanism of cysteine modulation. Consequently, high levels of free radicals can contribute to cancer by DNA damage and mutation mechanism but also, by increasing cellular growth rates affecting to proteins which control growth, survival and differentiation of cancer cells.

## 5. Antiproliferative and Pro-Apoptotic Activity of Antioxidants

### 5.1. Diet Antioxidants and Cancer

It seems obvious that if there is a redox control of cell growth, antioxidants might affect cellular growth either in physiological or pathological conditions. Two types of antioxidants must play a role in cancer, namely those exogenous antioxidants incorporated mostly by dietary intake but also, those endogenous antioxidants which contribute to control normal cellular redox status.

From a basic point of view, antioxidants are efficient inhibitors of cell cycle progression and cell proliferation. Since early 90s, the antiproliferative activity of antioxidants such as *N*-acetylcysteine has been described [96] but it was not until recently, that the way those molecules work on cancer cells was elucidated [97]. In fact, nice data provided by Goswami *et al*. found that G0/G1 transition to S-phase of the cell cycle, must be promoted by a transitional peak of superoxide radicals. Furthermore, antioxidants that depurate free radicals are able to retain cell in a resting state [97,98,99]. Thus, thiol antioxidants perturb cellular proliferation by regulating the cell cycle and redox state in cells. On the contrary, oxidants production after release from NAC-induced G0/G1 arrest is associated with entry of murine embryonic fibroblast into S phase.

Epidemiological studies have established a relationship between the incidence of cancer and consumption of certain types of food. The presence of antioxidants in diet has been directly related to lower incidence of cancer. In fact, chemoprevention has attracted the attention of oncologists and molecular biologists to modulate carcinogenesis. A chemopreventive agent can inhibit carcinogenesis either by blocking initiation or by stopping or reversing promotion and progression. Animal and cellular models have been used to discover a new class of chemopreventive agents, including flavonoids, polyphenols or isothiocyanates [100]. The active components of dietary phytochemicals that most often appear to be protective against cancer are curcumin, genistein, resveratrol, diallyl sulfide, S-allyl cysteine, allicin, lycopene, capsaicin, diosgenin, 6-gingerol, ellagic acid, ursolic acid, silymarin, anethol, catechins, eugenol, isoeugenol, dithiolones, isothiocyanates, indole-3-carbinol, isoflavones, protease inhibitors, saponins, phytosterols, inositol hexaphosphate, vitamin C, d-limonenen, lutein, folic acid, beta carotene, selenium, vitamin E, flavonoids and dietary fiber [101]. Most of them display antioxidant properties *in vitro* but also affect multiple signaling pathways including transcription factors activation, like NFκB or AP-1, pro-oxidant or pro-inflammatory proteins, like COX-2 or iNOS or antioxidant proteins such as glutathione peroxidase (Table 1).

**Table 1:** Natural antioxidants and their molecular targets implicated in cancer prevention.

Bioactive Substance	Source	Tumor	Molecular Pathways 1
Curcumin	*Curcuma longa* extract (turmeric)	Ovarian, prostate, oral, gastrointestinal tumors	NFKB1, AP-1, STAT1, STAT3, STAT5, CTNNB1, NFE2L2, IKBKB, EGFR, HER2, AKT, JNK, PKA, BCL2, BCL2L1, AR, TP53, GST, GPX, HMOX1, XOD, CCND1, ALOX5, PTGS2, NOS2
Genistein (isoflavone)	Soybeans, chickpea, kudzu root *Pueraria labata radix*; *Cicer arietinum*; *Desmodium uncinatum*	Ovarian, prostate, colon, breast	SLC2A1, ER, NFE2L2, autophagy, Multiple Tyrosine kinases, NFKB1, CASP12, CDKN1A, GPX
Resveratrol	Stilbene found in *Fabaceae* and *Vitis vinifera*	Prostate, breast, colorectal	PTGS2, NOS2, JNK, MEK, NFKB1, AP-1, CDKN1A, NFE2L2, TP53, BAX, caspases, BIRC5, CCND1, BCL2, BCL2L1, ALOX5, VEGF, AR, KLK3, HMOX1
Diallyl sulfide, S-allyl cysteine, allicin (garlic compounds)	*Allium* spp*.*	Hepatoma cells, Hematologic tumors, colon, neuroblastoma	IAP, Oxidative Stress generation, Cell Cycle arrest
Lycopene, beta carotene	Tomato, carrot, red fruits	Prostate	NFKB1
Capsaicin	Pepper, red chilis, paprika (*Capsaicum* spp.; *Euphorbia* spp.; *C. annum*; *C. frutens*)	Colorectal, gastrointestinal, nasopharyngeal	TRAIL, SP1, Cell cycle arrest, Apoptosis, NFKB1
Diosgenin	*Discorea* spp*.*	Prostate, Lung, Colon	HGF, TRAIL, MAPK1
6-Gingerol	Ginger, From *Zingiber officinale*	Lung, Hepatocarcinoma cells	Telomerase, NOS2, TNF1, APAF1, NFKB1, Caspases
Ellagic acid	Berries	Oral, breast, Prostate, Colon	CTNNB1, WNT, Apoptosis, AKT
Ursolic acid	Fruits, berries, aromatic herbs	Breast, Melanoma	NFKB1, BCL2, BCL2L1, BIRC5, TP53
Silibinin (sylimarin)	Milk thistle (*Sylibum marianum*)	Lung, leukemia, gastric	NFKB1, AP-1, MAPK, PTGS2, CCND1 EGFR
Anethole	Anise, fennel	Lung	AKT, NFKB1, MMP2/9
Catechins (flavonoids, flavanols)	Tea (*Camelia sinensis*)	Breast, prostate	NFKP1, AP-1, JNK, PGST2, CCND1, HMOX1, TP53, IGF, BCL2, CDKN1A
Eugenol, isoeugenol	Cloves (*Eugenia caryophyllus*)	Breast, cervix, colon	NFKB1, PTGS2, NOS2
Indole-3-carbinol (isothiocyanates)	Cruciferous (*Brassica* spp*.)*	Ovary, colon, lung, prostate	Telomerase, SP1, Cell cycle arrest
Saponins	Soybean (*Glycine max)*	Breast, skin, gastric	PTGS2, NOS2, MAPK, NFKB1, MMP
Vitamin C	Many fruits and vegetables	Colon, Ovary, prostate	Oxidative stress, Cell cycle arrest, HIF
d-Limonene	Citrus oils	Lymphoma, breast, gastric	PTGS2, NOS2, ERK, caspases
Lutein	Tomato	endometrial	
Folic acid	Leafy vegetables, beans, fruits	Colorectal cancer, neuroblastoma	
Selenium	Esophageal, breast, lung prostate, gastrointestinal		Co-factor of GPX, NFKB1, PTGS2, ER, TP53, CDKN1A
Vitamin E, tocopherols	Esophageal, breast, pancreas		NFKB1, PTGS2, NOS2, VEGF, AKT, ERK

^1^ Abbreviations used according to HGNC nomenclature: CTNNB1, β-catenin; survivin, BIRC5; NFE2L2, nuclear factor (erythroid-derived 2)-like 2 (Nrf2); IKBKB, IKK-Beta; BCL2L1, Bcl-XL; HMOX1, Heme Oxygenase 1 (HO-1); CCND1, Cyclin D1; ALOX5, 5-Lypooxygenase (5-LO), PTGS2, Cyclooxygenase-2 (COX2), SLC2A1, solute carrier family 2 (GLUT1); CDKN1A, P21/WAF1; KLK3, Prostate specific antigen (PSA).

In that way, vitamins and other antioxidants present in fruit and vegetable-rich diets have been demonstrated to be powerful inhibitors of cell growth *in vitro* and in *in vivo* models. As an example, vitamin C, which is mostly present in a reduced form as ascorbic acid in human body, has been shown to inhibit the growth of several cancer cells by decreasing oxidative damage caused by carcinogens [102] or by promoting cell toxicity [103]. Similarly, vitamin E has been employed in several cellular contexts to inhibit proliferation or promote differentiation of cancer cells. Vitamin E inhibits cancer cell growth either by decreasing cell proliferation in estrogen dependent breast cancer cells [104] or by increasing FAS-mediated apoptosis [105]. Also, lycopene and apo-12'-lycopenal reduce the proliferation of prostate cancer cells, in part, by inhibiting normal cell cycle progression and at low concentration and could synergistically inhibit the proliferation of colon cancer cells with eicosapentaenoic acid (EPA). The inhibitory mechanism was associated with suppression of phosphatidylinositol 3-kinase/Akt signaling pathway. Furthermore, treatment of lycopene and EPA also synergistically blocked the activation of downstream mTOR molecule [106].

Finally, several nutraceuticals including curcumin, resveratrol o genistein are able to reduce cancer cell proliferation by down-regulating the expression of cyclins or the activity of kinase dependent cyclins. In particular, cyclin D1 has been shown to be overexpressed in many cancer types including breast, esophagus, head and neck or prostate. In fact, resveratrol has been proved that exerts is anti-cancer effects by arresting cell cycle in colon adenocarcinoma cells, esophageal carcinoma cell, medulloblastoma cells, pancreatic carcinoma cells, human breast carcinoma, lung or leukemia cell lines. Resveratrol treatment caused an inhibition of cyclin D1 and cyclin-dependent kinase activity in all of them [101]. A direct relation between antioxidant properties of those compounds and their cell cycle inhibition is not clesar. However, considering that the transition from G0 to G1 is the only phase of the cell cycle which is not regulated by cyclin-dependent kinases, but rather by redox-dependent signaling pathways, expression of cyclin D1 represents a primary regulatory node for the dose-dependent effects of oxidants on the induction of cell growth. It was suggested that the expression of cyclin D1 represents a useful marker for assessing the integration of proliferative and growth inhibitory effects of oxidants on the redox-dependent signaling, and it seems clear when antioxidant compounds are employed to reduce proliferation [107].

### 5.2. Apoptosis Induced by Antioxidants

Free radicals and antioxidants affect cancer cells behavior in many ways, depending on cell type or redox intracellular state. Since radical species can increase proliferation, halt cell cycle, or induce either senescence, apoptosis or necrosis, antioxidants might inhibit cancer cells growth in many different ways too. Activation of apoptosis might account for antioxidants’ inhibition of tumor progression since a balance between cell growth and death is necessary in tissue homeostasis. Tumor cells display several mechanisms of survival including inhibitory mechanisms of apoptosis. Several reports showed that activation of transcription factor NFκB promotes cell survival and proliferation and its downregulation sensitizes cells to apoptosis [108]. NFκB controls the expression of several genes including Bcl-2, Bcl-X_L_, cIAP, survivin, TRAF1 and TRAF2, all them known inhibitors of apoptosis. Thus, those compounds able to inhibit NFκB nuclear traslocation or binding activity are demonstrated to induce apoptosis in tumor cells. Also NFκB is a redox sensitive transcription factor that it is inhibited by the treatment with antioxidants, including vitamins, scavengers or metal chelators [109]. Considering this, much investigation about the role of antioxidants in promoting tumor cell death has been focused in their role as inhibitors of NFκB activation. In general, numerous publications support the role of phytochemicals, such as curcumin, resveratrol, flavopiridol, betulinic acid, ursolic acid, indole-3-carbinol, zerumbone, green tea polyphenols, among others, in reducing the expression of apoptosis suppressor proteins such as Bcl-2, Bcl-X_L_ or survivin in cancer cells. Curcumin suppresses the constitutive expression of Bcl-2 and Bcl-X_L_ in mantle cell lymphoma or multiple myeloma cell lines [110]. In addition, curcumin also activates caspase-7 and caspase-9 and induced polyadenosine-5'-dephosphate ribose polymerase (PARP) cleavage in both cell lines. Also, it has been reported that resveratrol exerts anti-cancer effects by promoting apoptosis in different types of tumor cells. Resveratrol inhibits metastatic breast cancer cell line, melanoma, pancreatic, esophageal adenocarcinoma or colon carcinoma cells apoptosis by decreasing Bcl-2 and Bcl-X_L_ protein levels but also, by increasing apoptosis promoting protein Bax [111]. Also, green tea polyphenols are demonstrated that inhibit several types of tumors by increasing apoptosis. In prostate adenocarcinoma, EGCG induces stabilization of p53 and down-regulates NFκB which results in reducing Bcl-2 protein levels [112].

In summary, much of the chemopreventive activity of nutraceuticals is mediated by their role on redox sensitive transcription factors and upregulation of apoptosis pathways. In general, the constitutive activation of those AP-1 or NFκB, transcription factors is associated with a survival phenotype of cancer cells. In addition, anti-cancer treatments, including gamma-radiation, cytokines or chemotherapy, by increasing ROS inside cells, can activate redox-sensitive transcription factors that finally are able to promote the activation of survival signals that inhibit the end point search by tumor treatment. In that way, cancer cells develop those mechanisms to escape therapy and searching for compounds able to inhibit those mechanisms without compromising its effectiveness is of interest to oncologists. Consequently, antioxidants can be promising molecules to employ alone or in combination of conventional anti-cancer treatments. However, are those antioxidants able to discriminate between normal and tumor cells, are those molecules compromising effectiveness of treatment and many more questions are still in the mind of cancer biologists and physicians.

### 5.3. Human Studies with Antioxidants

Interestingly, several clinical trials have increased confusion about this topic. A good review of strong and weak aspects of those clinical trials has been made in a systematic review and meta-analysis conducted by Bardia *et al.* [113]. By using multiple electronic databases they search for randomized clinical trials to estimate the association between antioxidant treatment and cancer incidence and mortality. Although their study concludes that beta carotene supplementation appeared to increase cancer incidence among smokers, while vitamin E has no effect and selenium might have antitumor properties, it has several limitations that even the authors considered. The heterogeneous group of antioxidants, different combinations and dosages but also, some limitations of the design of the trial or the interpretation of results are important issues to be considered. In this regard, the expert opinion of Halliwell published in *The Lancet* in 2000 [26] about the use of antioxidants for the treatment of human diseases might be considered. In fact, some factors can help to understand contradictory results in cancer trials too. The protective effect of diet in cancer is not equivalent to a protective effect of antioxidants, and also, a good antioxidant might be a reducing agent depending on the environment in which it is working. Human cells are in a reduced state, but some degree of localized oxidation is needed. Thus, antioxidants can sometimes work differently depending on the redox situation inside cells. Similarly, in cell culture models we can find a molecule that can be a powerful antioxidant or a pro-oxidant agent, depending on cellular context. That is the case of vitamin C, which prevents the damage of the herbicide paraquat when it is employed prior to exposition to the toxic agent but it can even aggravate paraquat toxicity when it is given afterwards. Thus, the more reducing an antioxidant is the more problems it might cause. The study of precise molecular mechanisms is necessary to develop adequate protocols, the use of antioxidants in anti-cancer treatment needs a pro study of the role of antioxidants and free radicals in cancer biology in the context of *in vivo* experiments. Antioxidants might also inhibit the oxidant signaling mechanisms that cells use to adapt to free-radical insults, so a participation of endogenous antioxidant defenses in tumor survival is an important issue to take into consideration.

## 6. Redox-Related Enzymes and Cancer

### 6.1. Superoxide Dismutases and Cancer

Antioxidant enzymes that remove the intermediates of oxygen reduction, in particular superoxide dismutases (SOD), catalase and glutathione peroxidase (GPX) act by preventing oxidative damage together with noncatalytic antioxidants, including non-thiol compounds as vitamins or polyphenols and sulfur-containing compounds, like glutathione (GSH) and thioredoxin (Trx) [114]. Consequently, two different types of oxidative alterations, ROS overproduction or physiological mild oxidative unbalances are responsible for regulatory processes in cancer cells; therefore, as mentioned above antioxidants can play multiple roles in cancer initiation, promotion or progression.

Among major antioxidant enzymes inside cells are, in addition to glutathione cycle proteins, superoxide dismutase enzymes. There are two major forms, mitochondrial or manganese-containing SOD (MnSOD/SOD2) and cytosolic or copper-zinc containing SOD (CuZnSOD/SOD1). In particular, MnSOD KO mice do not survive long after birth due to severe lung damage and neurodegeneration. It was first observed that UV radiation stimulates MnSOD expression but also, that it mediated gene expression in radiation-induced adaptive responses [34]. Huang *et al*. reported that 2-methoxyestradiol (2-ME), an estrogen derivative that lacks the ability to bind to the estrogen receptor, kills human leukemia cells by promoting apoptosis. This apoptosis is mediated by inhibition of CuZnSOD which in turn promotes accumulation of O_2_^−^. In addition, overexpression of CuZnSOD reverted sensitivity to 2-ME but treatment with oligonucleotides against SOD were more sensitivity to 2-ME [115].

Differences in antioxidant status between normal and tumor cells have been found. Generally, lower levels of antioxidant enzymes have been found in tumor cells in early progression states. Thus, as an example, the expression of three major antioxidant enzymes, CuZnSOD, MnSOD and catalase were altered in human prostate carcinoma [116] which has been associated with higher sensibility to DNA damage cause by ROS. CuZnSOD, MnSOD and catalase had lower expression in high grade prostatic intraepithelial neoplasia (PIN) and prostate carcinoma than in benign epithelium. In the same way, cytosolic glutathione S-transferases (GSTs) which are a superfamily of enzymes that protect normal cells by catalyzing conjugation reactions of electrophilic compounds to glutathione, tends to decrease in some sort of cancer cells. Thus, it promotes the pro-oxidant environment necessary for progression, as it occurs in renal cell carcinoma growth [117]. Moreover, GST polymorphism are commonly found in other types of cancer, where its activity is compromised, including ovarian cancer or prostate [118]. Glutathione transferase GSTM1-null genotype may increase risk for tobacco related cancer through the impairment of polycyclic aromatic hydrocarbons detoxification.

In general, an increase in oxidative and decrease in antioxidant status has been observed in several types of tumors [119]. In addition, the overexpression of antioxidant enzymes has been an efficient strategy to reduce proliferation of normal but also cancer cells. MnSOD has been considered a tumor suppressor gene in several human and murine tumor cell lines. Thus, MnSOD overexpresion reduces the proliferation of melanoma, prostate, pancreatic, breast, oral carcinoma and glioma cellular growth [120,121,122]. Also, overexpression of CuZnSOD, reduces the growth of glioma [123]. The decreased rate of tumor cell growth was correlated with the enzyme activity ratio of CuZnSOD:GPx. Glioma cells that stably overexpressed CuZnSOD demonstrated additional suppressive effects on the malignant phenotype and changes in the cellular redox status, attributable to the accumulation of hydrogen peroxide or other hydroperoxides and, a possible reason to explain the suppression of tumor growth observed in CuZnSOD-overexpressing cells.

Many strategies of tumor cells to survive against anticancer treatments are related with the activation of NFκB activity. NFκB regulates, among others, the expression of some antioxidant enzymes including MnSOD. NFκB is also closely linked to MnSOD expression induced by phorbol myristate acetate, cytokine, and serum starvation leading to antiapoptotic responses to TNFα [124]. The role of MnSOD in cellular survival is clear, since downregulation of MnSOD compromises cell viability in several contexts. Chronic exposure of cells to ionizing radiation (IR) induces an adaptive response that results in enhanced tolerance to the subsequent cytotoxicity of IR and by using MnSOD knockout mice (Sod2^−/−^), MnSOD was shown to be essential in protecting against ROS-induced injury during O_2_ metabolism. Moreover, endogenous SOD activity is also involved in the resistance of Wilms' tumors to chemotherapeutic agents and in protecting human leukemic and cancer cells from radiation. The expression of MnSOD causes significant alterations in the malignant phenotype as well as inhibition of tumor growth *in vivo*. It was demonstrated that in MCF-7 cells treated with fractionated ionizing radiation or overexpressing MnSOD showed significant radioresistance, and endogenous MnSOD was induced in irradiated wild-type MCF-7 cells. A causal relationship between MnSOD expression, increased radiation resistance, and increased expression of stress-responsive genes and also, the inhibition of NFkB-mediated transcriptional activity with mutant IkB radiosensitized MCF7 cells treated with fractionated ionizing radiation as well as reducing the transcription of MnSOD [34]. Actually, our own results indicate that overexpression of MnSOD in prostate cancer cells is a common marker of neuroendocrine phenotype which is associated to a higher resistance to hormonal control and survival. That targeting antioxidant defense may be a promising approach to the selective killing of cancer cells. Mechanism based on combinations of cancer inhibitors with free-radical-producing agents may have clinical application [114,125].

Based on evidence, a unique anticancer strategy for treatment of tumors named “oxidation therapy” has been proposed involving inducing cytotoxic “oxystress”. This means that, mainly by using two methods, namely: (i) inducing the generation of ROS directly in solid tumors and (ii) inhibiting the antioxidant enzyme (defense) system of tumor cells, it would be feasible to promote cancer cell death [126,127]. Since the late sixties, the use of H_2_O_2_ for treating tumors in animal tumor models was assayed by injection in tumoral tissues or by administration into the circulation. Although, no satisfactory results were observed, these were the pioneering studies in using ROS as anticancer therapy. Side effects due to loss of selectivity were major problems. Today the anticancer potential of these agents is still under investigation, and it is intended to be solved by including macromolecular drugs, polymeric micelles or nanoparticles. In addition, downregulation or inhibition of antioxidant enzymes are expected to become anticancer strategies in the future. Heme oxygenase 1 (HO-1) [126] and MnSOD [115] play a central role in the defense in healthy tissues and become much important in tumors for defending against ROS and ROS-generating antitumor agents. The inhibition of HO-1 (see below) or SOD has been also proposed as an effective method for killing tumor cells. Then, antitumor strategies based on “oxidation therapy” might be a promising approach for clinical maneuver of tumors which are still resistant to conventional treatments.

### 6.2. A New Role of MnSOD in Cancer: Cell Differentiation

Some years ago, our laboratory started a new research line with the intention of using a combination of radiotherapy with the endogenous antioxidant melatonin to induce cell death in prostate cancer cells. Based on the rationale that, years before, it had been demonstrated that melatonin works as an efficient antioxidant and also, that melatonin inhibits NFκB activation after gamma irradiation or cytokine treatment [128], we studied its role in tumor growth and promotion of apoptosis. Androgen-independent prostate cancer cells are highly resistant to chemotherapy or radiation because they show a constitutive activation of NFκB and high levels of antiapoptotic proteins like Bcl-2 and survivin. Melatonin had been shown previously to reduce proliferation through its antioxidant activity [129], but its role in inducing apoptosis in cancer cells had not been explored at that time. Surprisingly, we found that this molecule was able to reduce proliferation, to inhibit radiation or TNFα-induced NFκB activation, but it was unable to promote apoptosis [125,130]. Moreover, cells treated with melatonin showed a neuroendocrine phenotype similar to other treatments described previously in the literature. The phenotype of neuroendocrine cells favors survival of prostate cancer cells and it is activated after several situations of cellular stress, including androgen withdrawal, IL-6 treatment or increment of cAMP intracellular levels. That surprise, lead our research to study the role of antioxidant defenses in prostate cancer cells survival. Years before, we also had found that melatonin regulates glutathione content and MnSOD, catalase or GPX mRNA, protein or activity [131]. Finally, we concluded that melatonin, and perhaps other antioxidants such as silibinin which also induce differentiation of prostate cancer cells, which do not increase apoptosis, but still inhibit NFκB activation, do not increase cell death because they are increasing endogenous antioxidants such as antioxidant enzymes or GSH [130]. Later on, we demonstrated that overexpression of MnSOD is a common mechanism during neuroendocrine differentiation in prostate cancer cells and it might be responsible for treatment resistance and cellular survival [132].

In that way, tumor cells develop many ways to escape cell death caused by cancer treatments. The design of cancer chemotherapy which avoids those mechanisms implicated in cancer cell survival is of interest. Resistance to treatment results from several factors, including individual variations in patients or somatic genetic differences in tumors. As therapy becomes more and more effective, acquired resistance is also a common event. The major players in cancer resistance to therapy is the expression of one or more energy-dependent transporters, insensitivity to drug-induced apoptosis, as mentioned above, or induction of drug-detoxifying mechanisms [133]. In those cases of resistance, the employment of multiple drugs with different mechanisms of entry into cells and different cellular targets allow for more effective chemotherapy and higher response. However, too often, cells express mechanisms that confer simultaneous resistance to many different structurally and functionally unrelated drugs. This phenomenon is known as multidrug resistance and results from changes that limit accumulation of drugs within cells by limiting uptake, enhancing efflux or affecting membrane lipids [134]. Once mechanisms of drug resistance were discovered, numerous strategies have been tested. Among others, targeting antiapoptotic proteins using antisense oligonucleotides strategies, including BCL-2, BCL-X_L_, or by enhancing IAPs activity by employing naturally occurring peptides, modulating methylation status of tumor suppressors or genes that affect drug sensitivity, are explored to favor drug sensitivity. In relation to antioxidant status, depletion of GSH to modulate drug resistance has also been considered [135].

### 6.3. Glutathione and Cancer

GSH is a cysteine-containing peptide that is synthesized by γ-glutamyl cysteine synthase (γ-GCS) in two steps. Intracellular concentration of GSH exceeds 10mM in some cells and it has been implicated in resistance to several chemotherapeutic agents. Increased levels of GSH are involved in resistance to platinum compounds in human ovarian carcinoma cells [136] but also as contributor to anthracycline resistance favoring its metabolism [137]. On the other side, depletion of GSH has also been demonstrated to increase the sensitivity to arsenic trioxide in multiple myeloma cells. Thus, retroviral vector-mediated overexpression of MRP1 and γ-GCS resulted in higher GSH levels and a greater level of resistance to sodium arsenite compared to overexpression of either MRP1 or γ-GCS alone. Then, GSH is an important cofactor of MRP1 conferring resistance to arsenical compounds [138]. Given that, three different strategies have been followed to deplete intracellular glutathione to increase sensitivity to therapeutic drugs. First, the employment of L-buthionine-(*S,R*)-sulfoxime (BSO), a potent inhibitor of γ-GCS has been demonstrated to deplete intracellular concentrations of GSH and enhance the sensitivity to platinum-containing compounds, alkylating agents, anthracyclines and arsenic trioxide [139]. Second, the use of a hammerhead ribozyme against γ-GCS mRNA to downregulate its levels specifically [140] and reversed resistance to cisplatin, doxorubicin and etoposide in HCT-8 cells colon cancer cells. Finally, a third strategy targeted c-Jun expression. Increased levels of GSH and γ-GCS were associated with a stable increase of c-jun expression and c-Jun binding to AP-1 transcription factor. By using, a 20-mer c-jun phosphorotionate ASO, a decrease of the steady-state levels of c-jun mRNA and protein was performed and a reduction in γ-GCS affecting GSH intracellular levels [136].

### 6.4. Nitric Oxide and Peroxynitrite

Nitric oxide (NO) has been one of those free radicals to be included in the list of cell signaling regulators in both animals and plants; even bacteria can sense very low concentrations of NO. As one of its discoverers has mentioned, the fact that NO is a free radical and can act as an intracellular messenger modulating enzymes such as certain isoforms of guanylate cyclase (GC), although now assumed, was initially viewed skeptically [141]. NO is produced from L-arginine by any of the three isoforms of NOS. NO then binds to the prosthetic heme group of NO-sensitive GC [142], which in turn activates this enzyme resulting in an increase in cGMP levels. This events lead to different cell responses including smooth muscle relaxation-essential for endothelium regulation- or neurotransmission. However, it is now widely accepted that, depending on the intracellular concentrations of NO reached, it may exert a variety of intracellular actions ranging from promoting cell survival, increasing cell proliferation or even inducing cell death [143].

Additionally, NO-derived metabolites regulate cell physiology at the protein level by formation of S-nitrosothiol, causing the so called S-nitrosylation of cysteine residues. This modification would act in a NO-mediated, cGMP-independent pathway, resulting in modulation of many key cell regulators including [144,145,146].

In addition to S-nitrosothiol, other NO-derived free radical species collectively called RNS are also formed, e.g., nitrosoium ioins (NO^+^), dinitrosyl-iron complexes and the combination of NO with O_2_^•−^, peroxynitrite (ONOO^−^). Peroxynitrite accounts for a rapid depletion of sulfhydryl groups and antioxidants, oxidation of lipids, DNA strand breakage, nitration and deamination of DNA bases and nitration of aromatic amino acids in proteins [147,148]. The most studied by far is the reaction with tyrosine residues to form 3-nitrotyrosine, leading in some cases to enzyme inactivation or to interference with signal transduction [149]. There is growing evidence about the potential role of 3-nitrotyrosine, at least as a biomarker, in cancer, but it still requires further research [150,151].

### 6.5. Xanthine Oxidase and ROS Production in Cancer

During purine catabolism, Hypoxanthine-Xanthine Oxidase (or Oxidoreductase, EC 1.17.3.2) (XOR) first catalyzes hydroxylation of hypoxanthine to xanthine and subsequently of xanthine to urate, giving rise to H_2_O_2_ as a byproduct. Since uric acid and its oxidation product allantoin may act as a free radical scavenger, XOR has consequently a pivotal role, either as an enzymatic antioxidant mechanism [152] or as a regulator of cellular redox status [153]. More recently it has been shown a role of XOR in innate immunity, as a mediator of infection and inflammation through its interaction with NF-κB and AP-1; furthermore it has also a role in ROS production during phagocytosis [154].

Nevertheless, several lines of research have shown the implication of XOR in different pathological situations through associated overproduction of ROS/RNS [155]. XOR has a role in ROS production during myocardial ischemia/reperfusion injury [156]. In this way microvascular endothelial cells preincubation with XOR increase adhesion of colon carcinoma cells [157]. Similarly, Griguer *et al.* have shown that XOR is one of the key enzymes producing ROS that stabilizes HIF-1α in cancer cells [158]. Interestingly, based on these and other evidence, some XOR inhibitors are under investigation for anti-cancer treatment [127,159].

### 6.6. Heme-Oxygenase 1

Heme-Oxygenase 1 (EC 1.14.99.3) (HO-1) is an inducible enzyme that catalyzes the rate limiting reaction in heme degradation to form CO, biliverdin and ferrous iron. While CO may serve as a second messenger in inflammation, proliferation and apoptosis [160], biliverdin is subsequently reduced to bilirubin and both of them have antioxidant properties. Finally ferrous iron induces the expression of ferritin, an important iron-storage protein. It is considered an antioxidant enzyme with anti-inflammatory, anti-apoptotic and anti-viral properties. Its expression remains basal under normal conditions but it is highly inducible as a response to several stimuli protecting cells against oxidative and inflammatory injuries. HO-1 deficiency has been reported in patients who died early with severe inflammatory symptoms [161,162], therefore revealing a key immunomodulatory role of HO-1. As it has been suggested by a body of evidence and epidemiological studies, chronic inflammation accounts for one third of all diagnosed cancers [163,164,165,166]. HO-1’s role in cancer displays a debate between the beneficial upregulation and subsequent protective role in spontaneous or induced autoimmune diseases and other chronic inflammatory pathologies against its harmful upregulation in tumor cells where the enzyme might even promote tumor growth by lowering the immune response. Recently, HO-1 upregulation has been associated to poor prognosis in lung cancer [167]. HO-1 also appears as a key upregulated gene in neuroblastoma [168], pancreatic [169], gastrointestinal [170] or colon cancer [171]. Targeting HO-1 with selective inhibitors has therefore become an important strategy in oncology.

## 7. Conclusions

**Figure 1:**
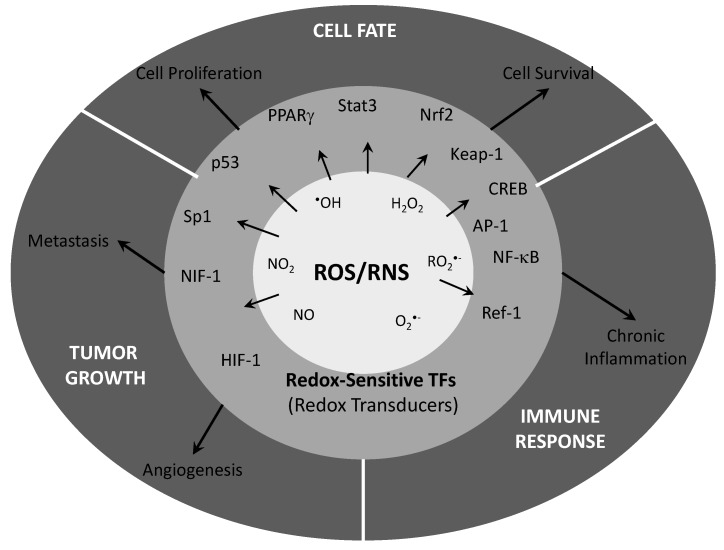
Oxidative species play an important role in cellular signaling through post-trascriptional modifications of important proteins. Those proteins modified by oxidative modifications show an important role in cellular process such as cell proliferation and survival. Both, deregulation of cell growth and death are the main aspects to explain cancer progression and promotion. ROS/RNS control by antioxidants has been efficiently proven to reduce tumor growth and progression both in cellular and animal models but a wide spectrum of biological player modify by oxidative species makes difficult to apply these knowledge to the clinical practice.

The fact that free radicals participate in tumor initiation by promoting DNA damage has been long accepted, but that same free radicals might take on an important role in signaling pathways has more recently been accepted (Figure 1).

The role of oxidative species as mediators of growth factors in several signaling pathways now seems clear, but their role in tumor progression it is still uncertain. It a role of oxidative species, such H2O2, in the activation of ASK-1 MAPK is also clear, but the participation of downstream cascades needs to be explored. Also, nuclear translocation and DNA binding of transcription factors, mainly AP-1 or NFκB, has been demonstrated, but their role in compromising survival of cancer cells through their activation is still unknown. In addition to other well-known mechanisms of drug resistance, adaptive mechanisms which imply overexpression of antioxidant enzymes have been demonstrated after irradiation in different types of tumor cells.

In addition to other principal players inside cells, high reactive molecules such as free radicals which are produced by cell metabolism, need to be carefully regulated to allow proper functioning of cells and a deregulation of those molecules and downstream signaling pathways might be a cause of pathological situations. Preliminary data, by using antioxidants *in vivo* clinical trials, have been contradictory respect to the use of these molecules as anti-cancer strategies, but abundant *in vitro* and *in vivo* evidence strongly support the role of free radicals, antioxidants and antioxidant enzymes in cancer initiation or progression. An effort should be made to find out the missing information in trials or aspects still unknown in the biology of tumor cells which might allow researchers and oncologists to find a better way to understand the scenario and the role that oxidative-mediate signaling plays in the disease.
